# Involuntary hamstring muscle activity reduces passive hip range of motion during the straight leg raise test: a stimulation study in healthy people

**DOI:** 10.1186/s12891-019-2511-6

**Published:** 2019-03-27

**Authors:** Yanni Foo, Martin E. Héroux, Lionel Chia, Joanna Diong

**Affiliations:** 1 Discipline of Physiotherapy, Faculty of Health Sciences, The University of Sydney, Lidcombe, NSW Australia; 2 Neuroscience Research Australia (NeuRA), Randwick, NSW Australia; 3 School of Medical Sciences, University of New South Wales, Randwick, NSW Australia; 4 School of Medical Sciences, Faculty of Medicine and Health, The University of Sydney, Rm 108, RC Mills Building (A26), Camperdown, NSW 2006 Australia

**Keywords:** Straight leg raise, Range of motion, Electromyography, Hamstrings, Passive

## Abstract

**Background:**

Involuntary hamstring muscle activity is present in some people during the straight leg raise test, but it is not known to what extent involuntary muscle activity limits passive joint range of motion. This study aimed to determine whether small amounts of involuntary hamstring activity limit passive hip range of motion during the straight leg raise test in healthy people.

**Methods:**

Thirty healthy subjects were recruited from The University of Sydney. As the hamstring muscles were continuously stimulated to generate 0, 2.5, 5, 7.5 and 10% of knee flexion maximal voluntary contraction force, an investigator blinded to the amount of stimulation performed a straight leg raise test by passively raising the tested leg while keeping the knee extended. The test was stopped when the knee started to flex, at which point hip range of motion was recorded.

**Results:**

On average, passive hip range of motion decreased by 0.6° for every 1% increase in knee flexion force caused by muscle activation (95% CI 0.3 to 0.9°, *p* = 0.0012). Subjects were instructed to fully relax when the straight leg raise test was performed, but a small amount of involuntary muscle activity (median 2.4% of maximal activation) was present during the trial without stimulation.

**Conclusions:**

Small amounts of involuntary hamstring muscles activity reduce passive hip range of motion during the straight leg raise test in healthy people.

**Trial registration:**

The protocol for this study was registered with the Open Science Framework, reference: https://osf.io/fejpf/. Registered 9 March 2017.

## Background

The passive straight leg raise test is popular among clinicians and investigators to quantify hamstring muscle extensibility, diagnose lumbar radiculopathy, and assess joint mobility [1–4]. During this test, the hip of a supine subject is flexed while keeping the knee extended. Hip flexion is stopped and range of motion is measured when the knee starts to flex, or when the subject reports discomfort in the hamstrings or lower back [3]. To ensure valid and reliable measurements, current best-practice protocols recommend that a known torque be used when hip joint angle is measured [5, 6]. Unfortunately, this is rarely if ever done in clinical practice.

Since muscle activity can limit passive joint range of motion, subjects should be fully relaxed when assessed. However, can subjects fully relax during the passive straight leg raise test? Results from healthy subjects are inconclusive. Involuntary hamstring muscle activity – 5 to 30% of maximal voluntary activation – has been reported in some studies [7–9], but not others [10, 11]. In line with these mixed results, Göeken & Hof [12] identified a sub-group of people with limited lumbar flexion but no neurological symptoms who have large amounts of involuntary hamstring muscle activity (45–100% of maximal voluntary activation) during the straight leg raise test. Critically, hip range of motion was reduced by approximately 30° in these patients.

Unsurprisingly, large amounts of involuntary hamstring muscle activity substantially reduce hip range of motion during the passive straight leg raise test. But, to what extent would small amounts of muscle activity reduce hip range of motion? Typically, involuntary muscle activity of less than 5% of maximal voluntary contraction (MVC) is ignored because it is assumed to be too small to reduce hip range of motion [13, 14]. However, if this assumption is incorrect, small amounts of involuntary hamstring muscle activity will limit, and potentially invalidate outcomes from the passive straight leg raise test. That is, reduced passive hip range of motion may be erroneously attributed to reduced passive hamstring muscle extensibility when, in fact, it is caused by involuntary hamstring muscle activity. Similarly, involuntary hamstring muscle activity and the associated reduction in hip range of motion could hinder therapists’ ability to elicit symptoms of lumbar radiculopathy during the passive straight leg raise test, complicating its diagnosis.

This study aimed to determine whether small amounts of involuntary hamstring muscle activity reduce passive hip range of motion during the straight leg raise test in healthy people. The hamstring muscles of healthy subjects were electrically stimulated to generate involuntary knee flexion forces ranging from 0 to 10% of MVC, while an assessor blinded to the amount of stimulation performed the straight leg raise test. We hypothesized that, even with these small amounts of involuntary muscle activity, hip flexion range of motion would decrease as hamstring muscle activity increased.

## Methods

This is an assessor-blinded, cross-sectional study. The procedures conformed to the Helsinki Declaration and were approved by The University of Sydney Human Research Ethics Committee (2016/748). The study protocol was pre-registered on the Open Science Framework (https://osf.io/fejpf/) and written consent was obtained from all subjects.

Thirty healthy subjects were recruited from The University of Sydney through poster advertisements and convenience sampling. Subjects were included if they were at least 18 years old and did not have previous injury or surgery to the right hip or knee. Subjects were screened to ensure there were no other factors (e.g. delayed onset muscle soreness) that prevented the straight leg raise test from being applied. The right legs of all subjects were tested. A sample size of 28 provided 80% power to detect a 5° mean change in hip angle (standard deviation 9°, alpha 0.05) [6].

### Protocol

**Fig. 1:**
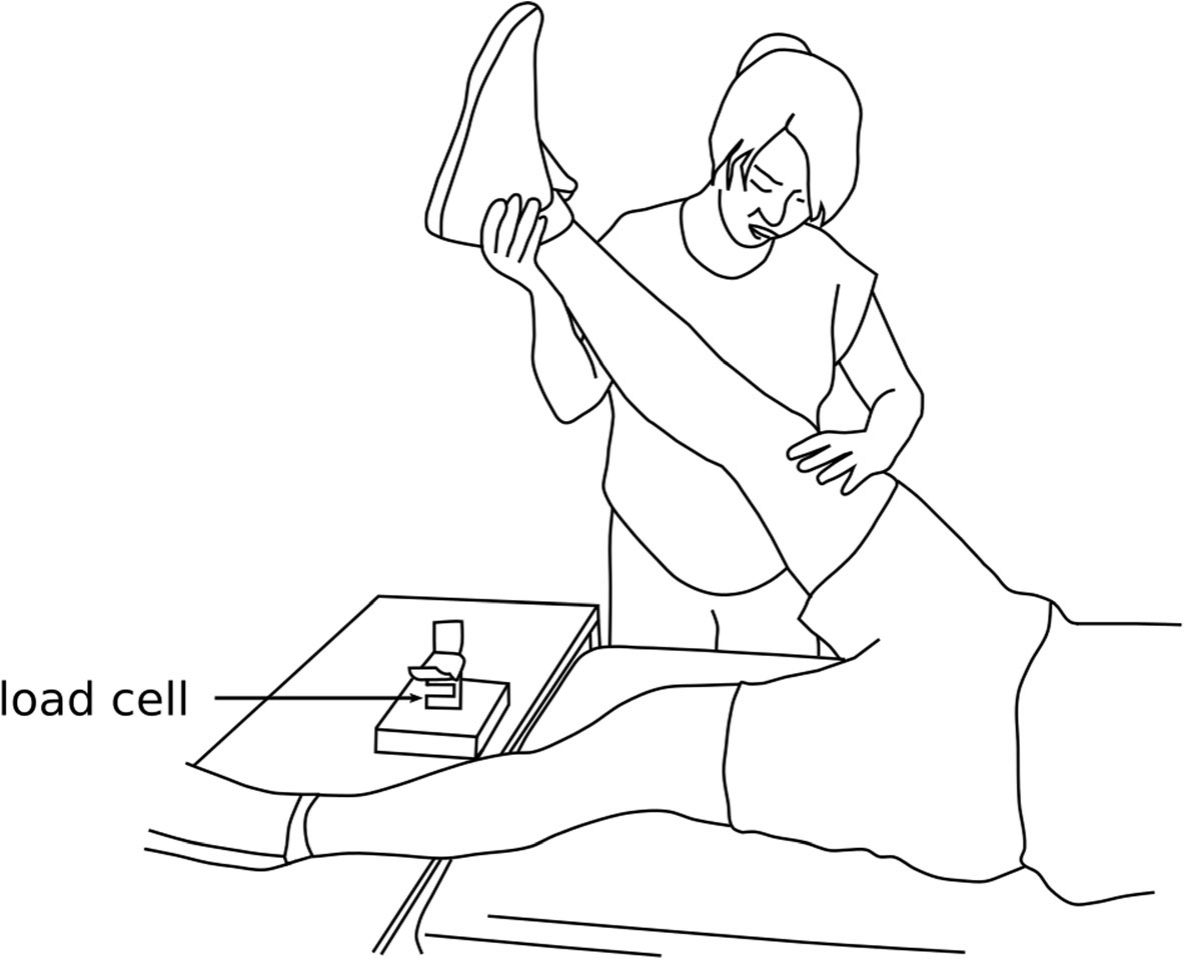
An investigator performs the passive straight leg raise test on a subject by flexing the hip while keeping the knee extended. The test is stopped when the knee starts to flex. Pairs of surface electrodes (not shown) were used to stimulate the hamstring muscles or record hamstring muscle EMG during knee flexion MVCs and during the trial without stimulation. The load cell was used to measure knee flexion MVC force

All testing was conducted during a single visit to a university laboratory. Subjects lay supine on a testing bench with their hips in a neutral position and the tested knee in ~ 5° flexion. The back of the ankle was placed on a load cell (XTRAN S1W 250 N; Applied Measurement, Melbourne, Australia) used to measure knee flexion MVC force (Fig. 1). The skin was cleaned and abraded with alcohol wipes before surface electrodes were applied. Two circular surface electrodes (diameter 10 mm, inter-electrode distance 30 mm) were applied to the back of the thigh over the muscle belly of biceps femoris. Two larger rectangular surface electrodes (50 mm by 90 mm, inter-electrode distance 60 mm) were also applied to the back of the thigh, proximal and distal to the circular electrodes. The hamstring muscles were stimulated through the large rectangular electrodes, while biceps femoris electromyography (EMG) signals were recorded through the small electrodes during knee flexion MVCs and during the trial without stimulation. A ground electrode was placed over the patella.

EMG signals were amplified and filtered (gain 200, 30–500 Hz band-pass; GRASS Model 15LT, Rhode Island, USA) before being sampled, along with the force signal, at 2000 Hz using custom-written LabView software (Labview 2013, National Instruments, Texas, USA) and a National Instruments data acquisition card (NI USB-6251, National Instruments, Texas, USA). A nine degree-of-freedom magnetometer (SparkFun SEN-10724, Colorado, USA) was attached to the distal tibia to measure hip joint angle.

Subjects first performed two isometric knee flexion MVCs against the load cell with the knee in ~ 5° flexion. Subjects were instructed to use only their hamstring muscles (not the gluteal or quadriceps muscles) to produce knee flexion maximal contractions. Verbal encouragement was provided during maximal contractions and a one-minute rest was provided between contractions. For each subject, peak forces during the MVCs were used to determine the intensities for continuous electrical stimulation required to achieve 2.5, 5, 7.5, and 10% of knee flexion MVC force. A constant current stimulator was used to deliver the continuous electrical stimulation (50 Hz, Model DS7AH, Digitimer, Hertfordshire, UK). The intensity required to achieve each target force was determined in separate trials, with a one-minute rest between trials. An additional trial without electrical stimulation (i.e. 0% MVC) was included. During this trial, the biceps femoris EMG signal was monitored to ensure subjects were as relaxed as possible.

The five stimulation intensities were applied to each subject in random order. Subjects were instructed to remain relaxed during all trials, and a one-minute rest was provided between trials. At the beginning of each trial, an investigator (LC or JD) started the electrical stimulation (or pretended to do so for the 0% MVC trial), then the blinded assessor (YF) performed a passive straight leg raise test. The right leg was raised while keeping the knee extended, the test was stopped when the knee started to flex, at which point hip range of motion was recorded (Fig. 1).

Hamstring muscle EMG signals from the knee flexion MVC and 0% MVC trials were digitally filtered (30–450 Hz, band-pass) and the root-mean-square EMG over a 50 ms window was calculated. EMG signals recorded during the 0% MVC trial were normalised to EMG at peak force during the MVC trial. Thus, involuntary hamstring muscle activity is reported as a percentage of maximal knee flexion activation.

### Statistical analysis

For each subject, linear regression was used to determine the effect of increasing knee flexion muscle activity (at 0, 2.5, 5, 7.5, and 10% of knee flexion MVC) on hip range of motion. Regression coefficients from all thirty subjects were then pooled to determine the overall mean effect (95% CI) of knee flexion muscle activity on hip range of motion. Secondary univariate sensitivity analyses were performed to determine whether age, sex or body mass index (BMI) had effects on hip range of motion. Data were processed and analysed using Python (version 3.5).

## Results

**Fig. 2:**
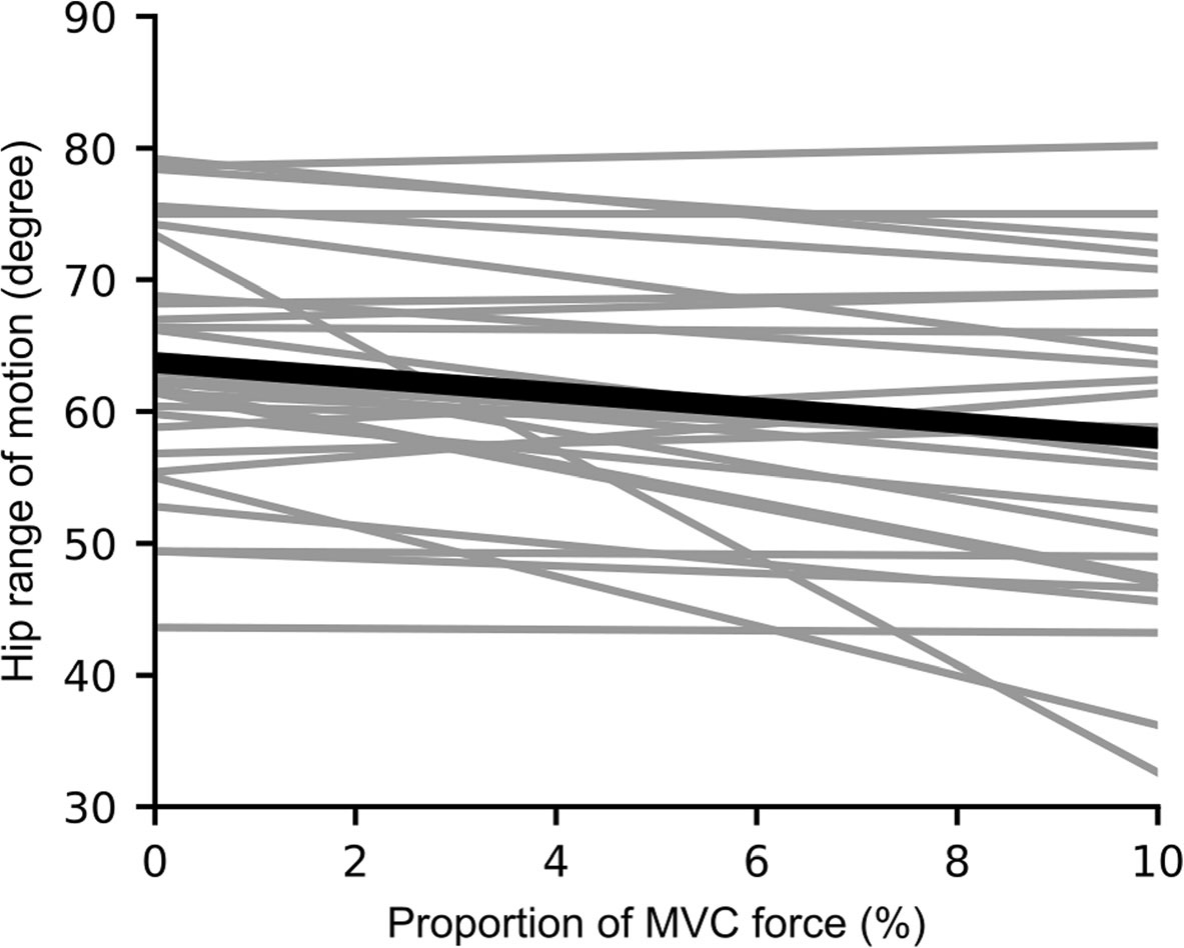
Linear regression of hip range of motion on proportion of knee flexion MVC force for each subject (grey), and the group mean (black)

Thirty healthy adults participated in this study; data are presented as mean (standard deviation, SD) except where indicated: age 23 (4) years; 12 males, 18 females; height 1.69 (0.10) m; weight: 63 (13) kg. On average, passive hip range of motion during the straight leg raise test decreased by 0.6° for every 1% increase in knee flexion MVC force (mean decrease in hip range of motion = 0.6°, 95% CI 0.3° to 0.9°, *p* = 0.0012; Fig. 2). In other words, hip range of motion decreased by approximately 2.9° on average when 5% of knee flexion MVC force was present. Across subjects, the median (interquartile range, IQR) hamstring muscle EMG during the 0% MVC trial was 2.4% (1.0 to 5.1%) of maximal activation. There were no effects of age (*p* = 0.45), sex (*p* = 0.60) or BMI (*p* = 0.20) on hip range of motion.

## Discussion

This study investigated the effect of small amounts of electrically-induced, involuntary hamstring muscle activity on passive hip range of motion during the straight leg raise test. Hip range of motion decreased by approximately half a degree for every 1% increase in knee flexion MVC force. This indicates that even at contraction intensities well below 10% MVC, hamstring muscle activity can limit results from the straight leg raise test.

Some points must be considered when interpreting the results of the present study. First, although subjects were instructed to fully relax when outcomes were measured, a small amount of involuntary muscle activity (median 2.4% of maximal activation) was present during the straight leg raise test. This additional muscle activity may have have caused the effect of muscle activity on reduced passive range of motion to be slightly overestimated. Second, the straight leg raise test was stopped when the knee starts to bend [3]. This stopping point is used in clinical practice and is subjectively determined, but hip range of motion measured at standardized torque may have produced slightly different results [6, 15]. During the straight leg raise test, the pelvis and contralateral knee were monitored for concomitant movement but were not stabilized to the testing bench. The tested leg was raised slowly by the blinded assessor at approximately the same rate for all trials and subjects. However, angular velocity and intra-tester reliability for the straight leg raise test were not formally assessed.

Our results indicate that clinical and laboratory studies that use passive range of motion outcomes measured without measuring muscle activity may be in error. For example, a within-subject randomized controlled trial found daily stretching did not improve ankle mobility in patients with spinal cord injury [5]. Compared to the contralateral ankle which served as the control, daily stretching did not improve ankle mobility measured at standardized torque. Although outcomes measured in the presence of “obvious electromyographic activity” were excluded, those measured in the presence of small amounts of involuntary muscle activity were likely retained. Based on our current results, inclusion of these measures would have reduced measures of ankle mobility, possibly masking a true treatment effect. The failure to consider the impact of involuntary muscle activity on measures of passive range of motion may explain in part why human studies on the mechanisms of contracture (i.e. loss of passive joint range of motion) are inconclusive [16–20]. Involuntary muscle activity is common during the passive straight leg raise test [7, 9, 12]. Randomized controlled trials have shown that stretching programs increase stretch tolerance (measured at maximal tolerated torque), but not muscle extensibility (measured at a standardized torque) [6, 15]. However, these and other studies [21–23] used the passive straight leg raise test to examine whether stretching is effective without considering whether involuntary muscle activity limits range of motion during assessment. Because of this, it cannot be determined whether stretching programs are ineffective, or whether real treatment effects are masked by the presence of involuntary muscle activity during outcome assessments or interventions.

Recently, a large systematic review of randomized controlled trials of stretch for contracture in neurological populations found no evidence of a clinically worthwhile effect (i.e. > 5° on average) [24]. However, only one trial [5] recorded muscle activity when outcomes were measured, so it is not possible to determine the extent to which results were affected by the presence of involuntary muscle activity. Indeed, it is recognised that contractures can be due to neurally and non-neurally mediated factors (e.g. spasticity) [25] and measuring passive range of motion at standardized torque “does not distinguish between the resistance caused by biomechanical and neural factors” [26]. Even if studies eliminated all outcomes measured in the presence of minimal to no muscle activity, what is the impact of involuntary muscle activity during stretching interventions? Can stretching increase muscle extensibility if administered in the presence of involuntary muscle activity? Large torque applied during the stretching intervention will likely overcome small amounts of involuntary muscle activity and provide a genuine stretch stimulus to the muscle. However the same point cannot be made for larger amounts of involuntary muscle activity. These are important questions that must be addressed to move forward. Specifically, future studies should determine the extent to which involuntary muscle activity limits the effectiveness of stretching and other physical interventions at increasing passive range of motion. This could be achieved by recording EMG while stretching, and including muscle activity as a covariate when assessing the effectiveness of stretching interventions. Alternatively, the effect of conventional stretching on passive joint range of motion could be compared to stretching while under electrical muscle stimulation at 5% MVC in a randomized controlled trial.

## Conclusions

The clinical implication of this study is that even small amounts of involuntary hamstring muscle activity can reduce passive hip range of motion during the straight leg raise test, a commonly-used clinical test. We recommend that clinicians and investigators record muscle activity when measures of passive joint range of motion are used to inform diagnoses or assess the effectiveness of interventions. The availability of low-cost, easy-to-use EMG systems make this recommendation increasingly feasible, even in the clinical setting. Importantly, our findings indicate that treatment effects based on passive joint range of motion outcomes require further investigation using accurate assessments of passive joint range of motion and concomitant muscle activity. More broadly, our results provide a new perspective from which to reassess previous findings on the effectiveness, or ineffectiveness, of stretching programs in both health and disease.

## Abbreviations

EMG: Electromyography
IQR: Interquartile range
MVC: Maximal voluntary contraction
SD: Standard deviation
